# Of Monkeys and Men: A Metabolomic Analysis of Static and Dynamic Urinary Metabolic Phenotypes in Two Species

**DOI:** 10.1371/journal.pone.0106077

**Published:** 2014-09-15

**Authors:** Edoardo Saccenti, Leonardo Tenori, Paul Verbruggen, Marieke E. Timmerman, Jildau Bouwman, Jan van der Greef, Claudio Luchinat, Age K. Smilde

**Affiliations:** 1 Laboratory of Systems and Synthetic Biology, Wageningen University and Research Center, Wageningen, The Netherlands; 2 Biosystems Data Analysis Group, University of Amsterdam, Amsterdam, The Netherlands; 3 Center for Magnetic Resonance (CERM), University of Florence, Florence, Italy; 4 Synthetic Systems Biology and Nuclear Organization Group, University of Amsterdam, Amsterdam, The Netherlands; 5 Heymans Institute for Psychological Research Psychometrics and Statistics, University of Groningen, Groningen, The Netherlands; 6 Nederlandse Organisatie voor Toegepast Natuurwetenschappelijk Onderzoek (TNO), Utrecht, The Netherlands; 7 Sino-Dutch Centre for Preventive and Personalized Medicine, Utrecht, The Netherlands; 8 Leiden/Amsterdam Center for Drug Research, Leiden University, Leiden, The Netherlands; 9 FiorGen Foundation, Florence, Italy; 10 Department of Chemistry, University of Florence, Florence, Italy; Steno Diabetes Center, Denmark

## Abstract

**Background:**

Metabolomics has attracted the interest of the medical community for its potential in predicting early derangements from a healthy to a diseased metabolic phenotype. One key issue is the diversity observed in metabolic profiles of different healthy individuals, commonly attributed to the variation of intrinsic (such as (epi)genetic variation, gut microbiota, etc.) and extrinsic factors (such as dietary habits, life-style and environmental conditions). Understanding the relative contributions of these factors is essential to establish the robustness of the healthy individual metabolic phenotype.

**Methods:**

To assess the relative contribution of intrinsic and extrinsic factors we compared multilevel analysis results obtained from subjects of *Homo sapiens* and *Macaca mulatta*, the latter kept in a controlled environment with a standardized diet by making use of previously published data and results.

**Results:**

We observed similarities for the two species and found the diversity of urinary metabolic phenotypes as identified by nuclear magnetic resonance (NMR) spectroscopy could be ascribed to the complex interplay of intrinsic factors and, to a lesser extent, of extrinsic factors in particular minimizing the role played by diet in shaping the metabolic phenotype. Moreover, we show that despite the standardization of diet as the most relevant extrinsic factor, a clear individual and discriminative metabolic fingerprint also exists for monkeys. We investigate the metabolic phenotype both at the static (i.e., at the level of the average metabolite concentration) and at the dynamic level (i.e., concerning their variation over time), and we show that these two components sum up to the overall phenotype with different relative contributions of about 1/4 and 3/4, respectively, for both species. Finally, we show that the great degree diversity observed in the urinary metabolic phenotype of both species can be attributed to differences in both the static and dynamic part of their phenotype.

## Introduction

In 2008, the first experimental evidence was presented that individuals of *Homo sapiens* species possess individual urinary metabolic profiles, as observed by means of nuclear magnetic resonance spectroscopy (NMR), allowing discrimination of individuals with near 100% accuracy [1]. The diversity observed in metabolic profiles of individuals is commonly attributed to the variation of intrinsic factors (such as (epi)genetic variation) and to extrinsic influences (such as diet habits, life-style and environmental conditions).

Nonetheless, the use of the individual metabolic phenotype as a tool towards improved personalized therapy and nutrition and enhanced pharmacometabonomics must rely on a deeper understanding of its building blocks.

To rationalize the makeup of the metabolic phenotype (*P*), it can be schematically viewed and approximated by means of a phenotypic equation as the summation of intrinsic factors (*I*), extrinsic factors (*E*) and their interaction (×) plus a residual part *R* not explained by the previous factors 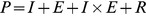
(1)


It is crucial to determine the relative importance of the different terms in Equation (1), but it is hard to study this in humans, as standardization of the environment is highly influenced by compliance of the subjects. While the influence of intrinsic factors is recognized and substantiated by several studies [2]–[4], the contribution of extrinsic factors on urinary metabolic profiles is still open to debate. Ludwig Feuerbach claimed that “*Man is what he eats*” [5], but it is unclear whether and to which extent diet and dietary habits can influence *P*.

Studies [6] and [7] report a strong association between diet and *P* whereas Winnike et al. [8] suggested the opposite, leaving the matter open for debate. New studies demonstrated that *P* is stable over a time period of at least three [9] to seven years [10] and have presented anecdotic evidence of this stability being independent of major life-style changes, including environment and dietary habits [9]. Here, stability refers to the concept that subjects can be uniquely identified after 2 to 7 years based only on their metabolic profiles, although the two studies utilized different statistical approaches.

However, in the course of studies [1] and [9], [10] it was not possible to derive a definitive conclusion about the role of dietary habits in making up *P*: in these studies the outcome was highly influenced by the compliance of the subjects to the standardized diet and other environmental parameters still differed between the subjects. Thus, the relative contribution of the extrinsic term *E* in Equation (1) could not so far be determined.

To overcome this limitation we took a comparative approach using high-level data fusion, *i.e*., applying identical statistical analyses on two different data sets. We compared results obtained for *Homo sapiens* (humans) with results of the analysis of urinary metabolic profiles of individuals of the species *Macaca mulatta* (monkeys) whose data were obtained from three previously published studies [1], [9], [11]. Subjects of the two species (31 humans and 10 monkeys) were sampled for their urinary profiles on 30 to 40 consecutive days and analysed by means of NMR. The two studies were identical in the experimental design but with one important difference: human participants were not restricted on extrinsic factors, whereas the monkeys were kept in a controlled environment and fed a standardized diet equal for all animals. We expected that when differences in *P* could be found for the monkeys these differences should be attributable to intrinsic factors as extrinsic factors were generally identical for all animals.

**Figure 1:**
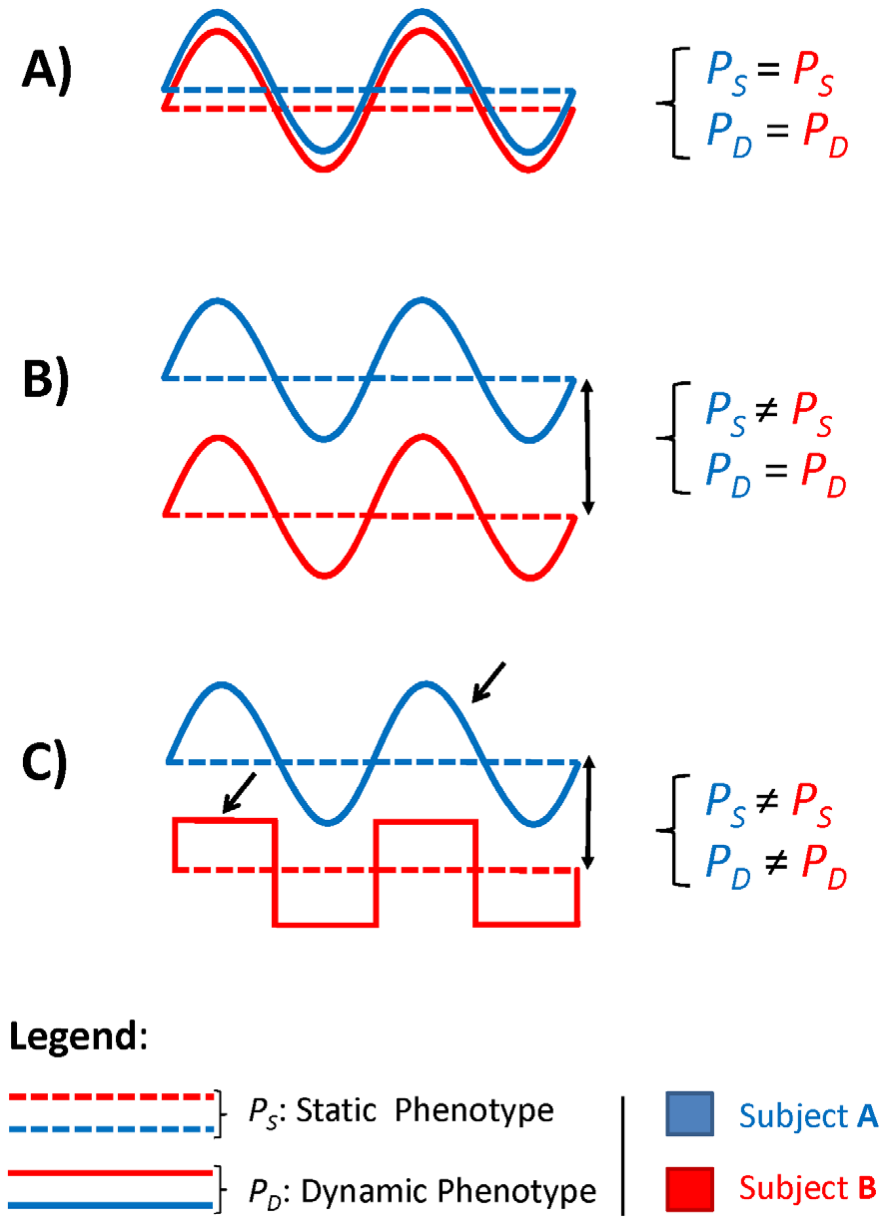
Partitioning of the individual metabolic phenotype in static and dynamic part. This cartoon introduces the concept of static and dynamic variation (i.e. static and dynamic phenotype). The dashed lines signify the average level concentration (of a metabolite), that is the static (*P_S_*) part of the metabolic phenotype. The solid lines signify the time dependent level concentration (of a metabolite), that is the dynamic part of the metabolic phenotype (*P_D_*). Taken together, the average concentrations of a metabolite and its modes of temporal variation constitute the metabolic phenotype (in this case mono-dimensional). Three cases are presented concerning two subjects, signified by colour blue (subject 1) and red (subject 2). Case A): Subject 1 and 2 are similar with respect to both the static and dynamic phenotype. Case B): Subject 1 and 2 are similar in the dynamic phenotype but different in the static phenotype. Case C): Subject 1 and 2 are different with respect to both the static and dynamic phenotype. The vertical double-pointed arrow (↕) indicates the difference of the average level (dashed lines) hence, the difference of the static phenotype. The single point arrow (↓) indicates the difference in the time profile shape (solid lines) and thus the difference of the dynamic phenotype.

To know the robustness of the individual metabolic profile, differences between *P* (either humans or monkeys) both at the statically (*i.e*. at the level of the average concentration of the metabolite in the urine), and also at the dynamic level (*i.e*. concerning their variation over time) should be analysed. Figure 1 exemplifies a one-dimensional representation (i.e. one metabolite) of the metabolic phenotype, where each individual evolves dynamically around an attractor defined by the average concentration level of that metabolite. These patterns of variation are well known in physiology: the observation of daily variation of potassium content in urine dates back to the nineteenth century [12], [13] and hormonal secretion has been shown to follow well defined circadian rhythms [14], [15]. On the basis of these observations, one can re-write Equation (1) making explicit the decomposition of the metabolic phenotype in a static phenotype *P_S_* and in a dynamic phenotype *P_D_* in such a way that 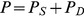
(2)Additionally, assuming that both the static and the dynamic phenotype are given by the summation of intrinsic and extrinsic factors (and their interactions) as already indicated by Equation (1), we can write:
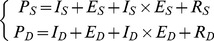
(3)


By means of a novel chemometric technique called Multilevel Simultaneous Component Analysis [11], [16] (hereafter termed multilevel analysis) we were able to quantify the approximate relative contribution of both *P_S_* and *P_D_* to *P* and, more important, we could highlight the source of variation leading to different *P_S_* and *P_D_* between subjects.

We found substantial overlap between the results obtained for human and monkey data sets, revealing similarities of individual urine metabolic phenotypes of both species. We observed that *P_S_<P_D_* for both *Homo sapiens* and *Macaca mulatta*, and that the building blocks of the static and dynamic part of the phenotype are linked to the same fundamental metabolic pathways for both species with likely negligible contributions from extrinsic factors. Additionally we provide evidence that discrimination among different monkeys, kept in standardized conditions, is possible with a near 100% accuracy, similarly to what was previous observed in the case of humans [1].

## Materials and Methods

### Sample and metadata collection

#### Human data

About 40 urine samples (first in the morning, preprandial) were collected from 31 healthy individuals (14 males, 17 females, all subjects were Caucasian) in the age range 25–55 over a period of about 3 months, in late-spring−early-summer of 2005 and 2007.

Subjects, all resident in the Florence area (Italy), were enrolled on a voluntary basis with age (>18 years) and absence of (evident) illness or disease as the sole exclusion criteria. A table with some anthropomorphic characteristics of the participants is given in the Table S1 in File S1.

Samples were collected from each individual in sterile 15-mL propylene tubes, frozen within 4 h of collection, and stored at −80°C. Personal data were collected from every subject, including gender, age, body mass index, and general habits such as practiced physical activity and normal diet. A detailed diet sheet relative to the day before each collection was also provided by each donor.

Due to the absolute non-invasiveness of the sample collection and to the fact that participation was on a voluntary basis ethical approval was neither needed nor requested at the time of the collection (2005–2007). Informed written consent was obtained [1], [9] from all participants. Data were anonymized and anonymously analysed.

#### Monkey data

Young adult, healthy rhesus monkeys (5 males and 5 females) (*Macaca mulatta*), were purchased from the Animal Science Department of the Biomedical Primate Research Centre (BPRC) in Rijswijk, The Netherlands. During an experiment the animals were individually housed. Each animal was identified by a tattoo on the chest. The standardized diet for the animal consisted of AM-II food-pellets (Hope Farms, Woerden, The Netherlands), rice, vegetables and fresh fruit. Drinking water was provided *ad libitum*. The diet was the same for all the animals. Environmental and cage enrichment was provided.

In accordance with the Netherland's Law on animal experimentation, study protocol involving living animals was reviewed and approved by the Biomedical Primate Research Centre's ethics committee. Experiments were performed in accordance with ethical guidelines of the Biomedical Primate Research Centre in Rijswijk.

Monkey urine samples were obtained at 30 days per individual. Urines were collected overnight in a fine-maze covered tray placed under the cage. After precipitation of debris by centrifugation the clear urine samples were decanted and stored frozen at -20°C until analysis [17].

### Sample preparation

Frozen samples were thawed at room temperature and shaken before use. Aliquots of each human urine sample (630 µl) were added to 70 µl of sodium phosphate buffer (0.2 M Na_2_HPO_4_ and 0.2 M NaH_2_PO_4_ in 100% 2H_2_O, pH 7.0) supplemented with10 mM sodium trimethylsilyl [2,2,3,3-2H4]propionate (TSP) and 30 mM sodium azide.

Monkey urine samples were lyophilized and pre-treated by adding 1 mL of urine to 1 mL of sodium phosphate buffer (0.1 M, pH 6.0, made up with D2O) containing 1 mM TSP as an internal standard (δTSP = 0.0).

The two sample preparation protocols are discussed further in the Note S1 in the File S1.

### NMR experiments

Human samples were measured using a Bruker 600 MHz spectrometer (Bruker BioSpin) operating at 600.13 MHz proton frequency. The 1D ^1^H-NMR spectrum of each sample was acquired with water peak suppression pulse sequence (NOESYGPPR1D; Bruker), using 64 free induction decays (FIDs), 64k data points, a spectral width of 20.0306 ppm, a relaxation delay of 4 *s*, and a mixing time of 100 *ms*. The FIDs were multiplied by an exponential weighting function corresponding to a line broadening of 1 Hz before Fourier transformation, phasing, and baseline correction.

Monkey NMR spectra were measured with a Varian Unity 400 MHz spectrometer. FIDs were recorded as 64k data points with a spectral width of 8.000 Hz. A single 45° pulse was used with an acquisition time of 4.10 *s* and a relaxation delay of 2 *s*. The spectra were acquired by accumulation of 128 FIDs. The signal of the residual water was removed by a pre-saturation technique in which the water peak is irradiated with a constant frequency during 2 *s* prior to the acquisition pulse. An exponential window function with a line broadening of 0.5 Hz and a manual baseline correction were applied to all spectra.

### Data reduction and pre-processing of the ^1^H-NMR spectra

^1^H-NMR spectra from all samples of both humans and monkeys were normalized to the total spectrum NMR signal intensity. After scaling, bucketing was applied to the data where the spectral regions δ>9.5, δ = 6.0–4.5, and δ<0.5 were discarded before dividing the remainder of each spectrum into sequential segments (“bins”) of 0.02 ppm width and obtaining an integral for each segment.

### Statistical analysis: PCA-CA KNN

Principal Component Analysis (PCA) on the model data were initially applied as in the PCA/CA/K-NN approach with purpose of dimension reduction. Multivariate analysis of variance (MANOVA) and CA were then applied to the model set representations in the relevant PCA subspace to define the subspace with optimum group separation. Test sets were first projected in the discriminating subspace defined by the model set and then the K-NN classification was applied. See [1], [9] for full details. Significance was assessed by means of permutation tests [18].

The PCA/CA approach may suffer, in principle, from the drawback that the sources of variation are mixed by the initial PCA dimension reduction. Nonetheless, as PCA is used as a dimension reduction technique and the original data are projected onto a subspace accounting for 99.9% of the variance of the original data, the data structure is preserved. A possible limitation of PCA-CA-KNN is that the discrimination procedure is in the CA space rather than in the metabolite space, hindering the interpretation of the metabolic profiles. Nevertheless, further analysis was performed to assess whether similar results as obtained with this technique in [1], [9] on the human data set, could be found for the monkey data set.

### Statistical analysis: multilevel simultaneous component analysis

Multilevel Component Analysis: Both the *Homo sapiens* and the *Macaca mulatta* data sets are two-level data sets, where urine samples are collected at different measurement occasions (level 1) for different subjects (level 2). Each data set contains different types of variation originating from static differences between subjects which are constant in time (like gender and genotype), and from dynamic differences which are subject specific (like biorhythms) [11]. To disentangle those sources present in those hierarchically ordered data, multilevel component simultaneous analysis (MSCA [11], [16]) is a suitable approach (here after multilevel analysis). The two-level MSCA applied here provides a model containing independent sub-models describing the two sources of variation, *i.e*., within and between subjects, related to the terms *P_D_* and *P_S_*, respectively, constituting Model (2) illustrated in the Introduction with Equation 2. For multilevel data, the MSCA models are easier to interpret than regular PCA models. The time-resolved variation of all subjects is expressed in the same subspace. The method is illustrated in detail in [11]. A brief outline is given below. MSCA is a component model, in which a simultaneous component analysis (SCA) model describes the within-group variation and a PCA model describes the between-group variation. The MSCA model is as follows (bold font signifies matrix and vectors, italic font signifies scalars):
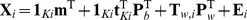
(4)where **X**_i_ is the data matrix of size *K_i_×J* pertaining to the *i*-th of *I* subjects, containing *K_i_* observations of *J* variables (NMR peaks/metabolites in the present case), **1**_Ki_ is a column vector of ones of size *Ki×1* and **m** is the row mean vector of **X**_i_. The between-subject scores for subject *i* are contained in the row vector **t** ^T^_b,i_ of size *R_b_*. The between-subject loadings are collected in the *J×R_b_* matrix **P**_b_, where *R_b_* is the number of components chosen to fit the between-subject model (1≤*R_b_*<*I*). **P**_w_ is a *J×R_w_* matrix containing the loadings for the within-subject model. The loadings are the same for all the subjects: this means that the scores for the within-subject model, contained in the *Ki×R_w_* are expressed in the same base and thus are directly comparable.

Two model parts can be distinguished: **B** = **T_b_P_b_** ^T^+**E**_b_ for the static (*i.e*. between) variation and **W** = **T_w_P_w_** ^T^+**E**_w_ for the dynamic (i.e., within subject) variation. In the MSCA model (1) the differences between subjects are explained by the term **1**_Ki_ **t** ^T^_b,i_ **P** ^T^_b_ which is different for different subjects. The variation of features within each subject is described by **T**_w,i_ **P** ^T^_w_.

A feature of the MSCA modeling exploited in this study, is that the information (variation) in the data set **X** can be split (and quantified) in its dynamic (*i.e*, within part) and static (*i.e*., between) in an ANOVA-like fashion [19] with the simple formula:
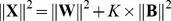
(5)where *K* is the number of observations for each subject. The variance explained for both models (i.e., how much of the static/dynamic information is accounted for by the model) is calculated analogously as in the standard principal component analysis (for more details see sections 2.6 and 2.7 in [11]).

In a MSCA model these two kinds of variation are modeled separately and are not confounded: this greatly improves the interpretation. The MSCA models are interpreted in terms of loadings and scores, as in the usual PCA model. The optimal numbers of between- and within-components to be fitted were determined by means of a scree plot [20]. The between- and within-components of a MSCA model can be plotted (in component plots) and interpreted as is usually done in PCA. In the between-component plot, each subject is represented in the space by two (or more) coordinates along the first two (or more) principal components. As each coordinate is a combination of the original variables (i.e., the metabolite concentrations in urine), subjects that are spatially close show similarity in their urinary profiles. Each component is a linear combination of the original variables: the loadings provide the weights that define the relative contributions of each variable to a given principal component, or, as used in the text to avoid too technical jargon, to provide a measure of the relative importance of a given metabolite to the model. Analogously, a within-component plot can be made for each subject, representing the measurement occasions in the space by coordinates along the (within) principal components.

Multilevel analysis is an extension of PCA and has the property that variables showing higher variability are stressed. When analysing raw data this may mask interesting biological phenomena. Therefore, data were Pareto scaled (i.e., each variable was centred around its mean and scaled over the square roots of its standard deviation [21]) to ensure homogeneous dynamic ranges across all buckets in the spectra.

### Statistical analysis: calculation of confidence intervals

To assess the inferential properties of the model estimates, and judge the generalizability of the results of MSCA, we estimated confidence intervals (CIs) with a bootstrap technique [22], a technique hitherto never applied in the context of multilevel modeling of metabolomics data. Bootstrapping requires a proper resampling scheme which in turn depends on which level(s) are considered as random and which level(s) as fixed. Though we would like to generalize across sampling occasions (level 1) and subjects (level 2), the sample size at level 2 is too small to estimate reliable CIs [22], and thus prohibits treating the subjects as random. With 20 level 1 units reasonably reliable 95% CIs around loadings can be obtained [22], which is satisfied for both human and monkeys data. Therefore, we treated level 2 as fixed, and level 1 as random in our bootstrap scheme [22]. Results (scores and loadings) are presented with their associated 95% CIs.

A list of all abbreviation used in the paper can be found in Table S3 in File S1.

## Results

In our analysis we exploited the fact that both data sets contain multiple samples collected sequentially over time for several subjects. This kind of data is said to contain multilevel information because it contains information about different sources of variation [1], [9], [11], in the present case static and dynamic differences among individuals/monkeys.

We applied multilevel analysis to model multilevel data; this novel chemometric technique returns two different models describing separately the static and the dynamic information, while retaining ease of interpretation. Moreover, for the sake of generalizability, we coupled it with an advanced statistical validation methodology based on bootstrapping [22], which allowed us to infer the subject-specificity of the metabolic urinary phenotype at a 95% confidence level.

Multilevel analysis was applied on the full bucketed NMR urinary profiles carrying information on hundreds of low molecular weight molecules, which mainly represent the byproducts of central metabolism and dietary intake. By means of multilevel analysis we were able to quantify the relative contributions of both the static and dynamic parts to the overall metabolic phenotype and, more importantly, we could highlight the sources of variation responsible for static differences between subjects (*P_S_*) and their individual dynamics (*P_D_*).

### Multilevel analysis of *Homo sapiens* urinary metabolic profiles

For *Homo sapiens*, 24% of the observed variability of measured urinary metabolic phenotypes is attributable to static variability, *i.e*. to *P_S_*_._ The remaining 76% is due to differences in the dynamic variation i.e. to *P_D_*.

The multilevel model was able to explain 81% of the subject-specific phenotype static diversity and 72% of observed variability in the dynamic phenotype. A summary of fit measures from the multilevel analysis is given in Table 1.

**Table 1:** Summary of the multilevel model for the static and dynamic phenotype.

	*Homo sapiens*	*Macaca mulatta*
**Static phenotype**	24%	24%
**Dynamic phenotype**	76%	76%
**Static phenotype diversity explained**	81%	77%
**Dynamic phenotype diversity explained**	72%	66%
**Dynamic phenotype diversity explained per individual**	30%–91%	49%–77%

**Figure 2:**
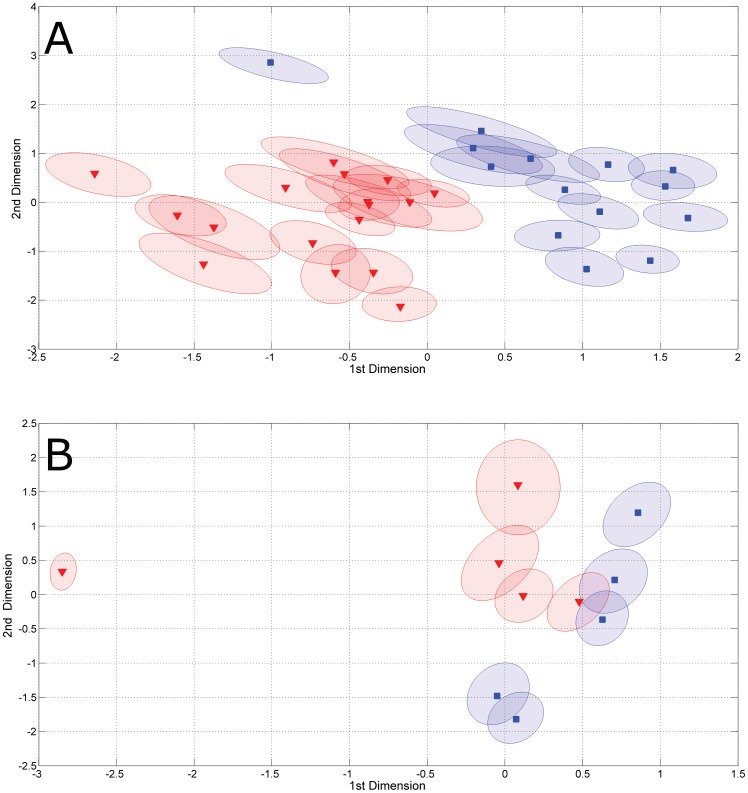
Multilevel model for the static phenotype *P_S_*. Two-dimensional plot of the multilevel model for the static phenotype. (**Panel A**: *Homo sapiens*; **Panel B**: *Macaca mulatta*). Each ellipsis envelopes the space of 95% CIs estimated by bootstrapping. Male subjects are color coded in blue (▪), female subjects is color coded in red (▾).

In the multilevel model for the static part of the phenotype, each individual is collapsed into a single point in a lower dimensional space able to capture (dis)similarities between the static phenotype of different subjects. Stated otherwise each dot represents the static phenotype of a different subject. Figure 2 (Panel A) shows the first two dimensions of the static model for each individual with its associated 95% confidence ellipse; there is relatively little overlap between different subjects, indicating that *P*_S_ is a subject-specific characteristic.

**Figure 3:**
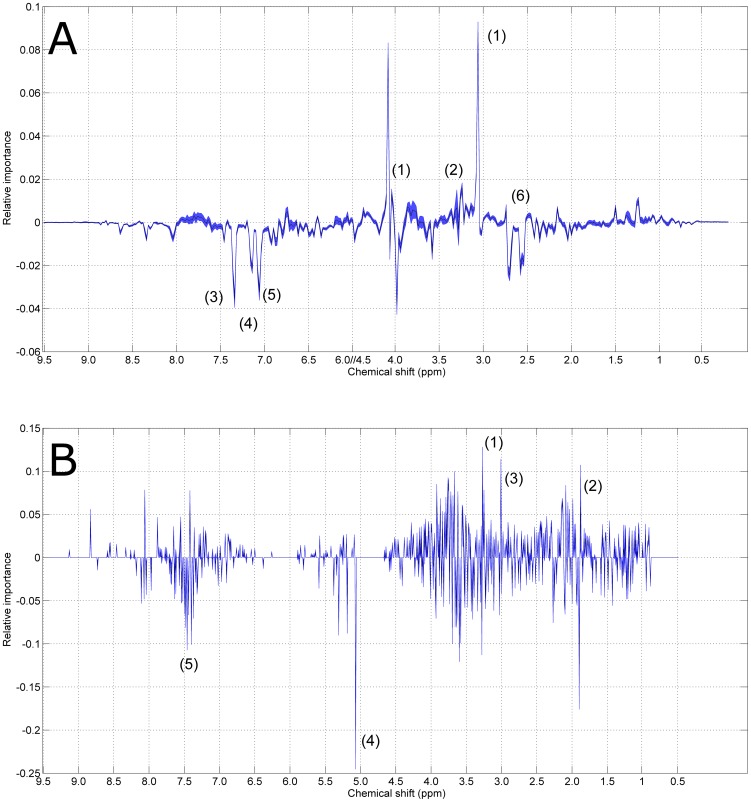
Metabolite relative importance to the model for the static phenotype *P_S_*. The shadowed area defines the 95% CIs estimated by bootstrapping (see **Figure 4** for a zoom-in). The resonances associated to the most (relatively) important metabolites are given below. **Panel A**: *Homo sapiens*. (1) creatine/creatinine, (2) TMAO, (3) phenylacetylglycine, (4) mHPPA and (5) 1-methylhistidine, (6) n-methylamine. **Panel B**: *Macaca mulatta*. (1) (2) acetate, (3) creatine/creatinine, (4) unassigned (5) indoxyl sulphate, PAG, hippurate.

**Figure 4:**
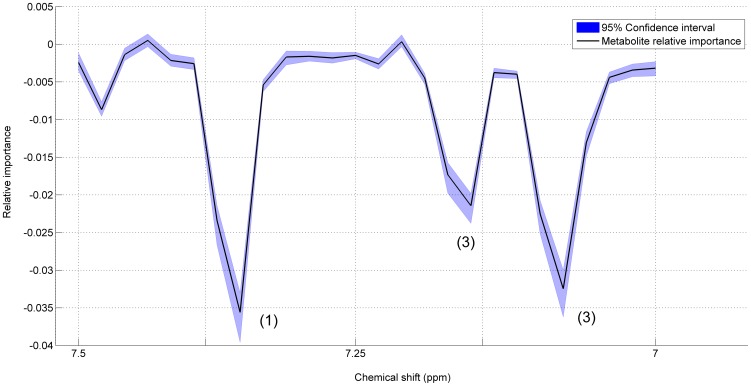
95% Confidence intervals. Zoom of the 7.5–7.0 ppm region for the model for the static phenotype for *Homo sapiens*. The shadowed area represents the 95% CIs for the given loadings (plotted in black). The (relative) importance associated to the resonances of phenylacetylglycine (1), mHPPA (2) and 1-methylhistidine (3) are shown.

The relative importance of each metabolite contributing to the static model (see Equations (2) and (4)) is described by the associated loadings which are mainly dominated by the resonances attributable to Trimethyl-*N*-oxide (TMAO), creatinine, phenylacetylglycine, **meta-hydroxyphenyl-propionic acid** (mHPPA) and 1-methylhistidine (see Figure 3, Panel A). Loadings are presented with the associated 95% CI's, obtained by bootstrapping; CI's are extremely narrow: a zoom of the region 7.5–7 *ppm* for the loadings of *P*_S_ is given in Figure 4. This indicates that, at a 95% confidence level, the loadings are the same for all subjects.

**Figure 5:**
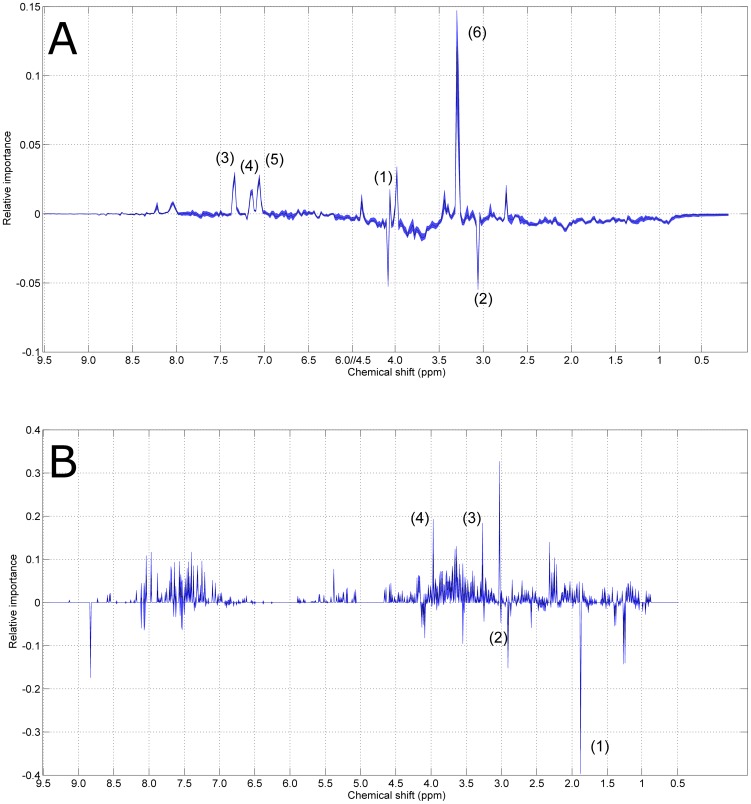
Metabolite relative importance to the model for the dynamic phenotype *P_D_*. The shadowed area defines the 95% CIs estimated by bootstrapping. The resonances associated to the most (relatively) important metabolites are given below. **Panel A**: *Homo sapiens* (1), creatine, (2) creatinine, (3) phenylacetylglicine, (4) mHPPA, and (5) 1-methylhistidine (6) TMAO. **Panel B**: *Macaca mulatta*. (1) acetate, (2) creatine/creatinine, (3) TMAO (4) hippurate.

The model for the dynamic phenotype is dominated by the resonance of TMAO as shown in Figure 5, panel A.

**Figure 6:**
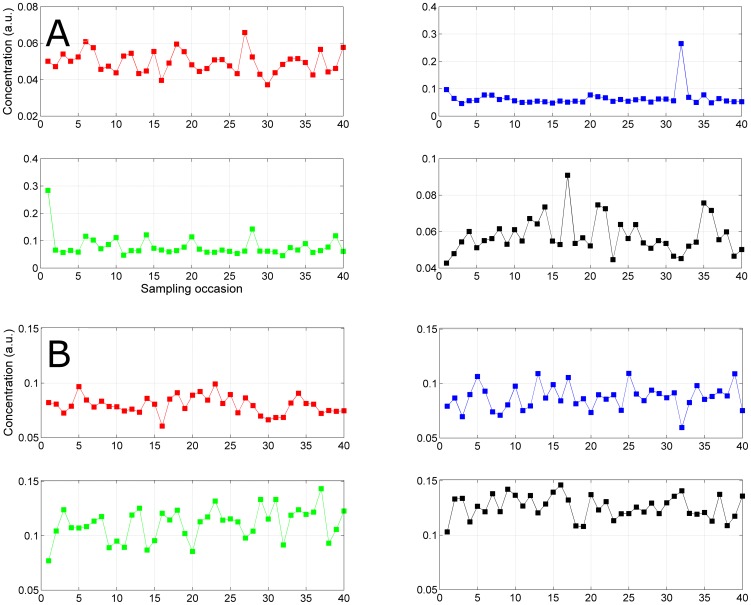
Subjects specific dynamics of TMAO and creatine. Dynamics of TMAO (panel A) and creatine (panel B) concentrations (expressed in arbitrary units) for four different individuals. While creatine shows similar scattered dynamic for all subjects, TMAO dynamics can be rather different between individuals.

Urine metabolite concentrations show a large degree of variability in the dynamic range, which furthermore varies between metabolites. For instance, the dynamic range of TMAO is much larger than those of creatine: the averaged (over the 31 human subjects) coefficient of variation of TMAO is 4 times larger than that of creatine (0.42 *vs* 0.12). Figure 6 shows the different dynamics of TMAO and creatine (panels A and B respectively) for four different individuals, giving a real life example of inter-individual difference of *P_D_*'s.

### Multilevel analysis of *Macaca mulatta* urinary metabolic profiles

In the case of *Macaca mulatta* 24% of the observed variability of the measured *P* is attributable to *P*_S_, while 76% is due to differences in *P*_D_, displaying a striking similarity to humans.

Figures 2 and 3 (Panels B) show the plots for the first two components of the static model, and the relative importance of the associated metabolites. Resonances attributable to TMAO, creatine, creatinine and acetate, fructose and an unassigned resonance at 5.07 ppm dominate the loadings, also in the case of the dynamic model as shown in Figure 3 (top).

The multilevel model was able to explain 77% of the difference between the static difference between subjects and 66% of the within individual variability. The percentage of dynamic variation explained per individual ranges between 49% and 77%. These numbers are summarized in table 1.

### Predictive analysis of *Homo sapiens* and *Macaca mulatta* data

Studies [1], [10] report that statistical analyses performed on NMR spectra of human urine samples reveal an invariant metabolic fingerprint characteristic of each person [1]. Using this fingerprint it is possible to correctly classify individuals with an overall accuracy approximating 99%. Moreover, *P* is relatively stable over a period of up to 2 to 7 years [2] [10]. When the same predictive analysis (using the PCA-CA-KNN approach as detailed in the Material and Methods approach) was applied we found correct classification rates of different monkeys varying between 85.6% and 100% (see additional table 1).

## Discussion

### Multilevel analysis highlights patterns of similarities in the urinary phenotype of *Homo sapiens* and *Macaca mulatta*

The constituents *P_s_ and P_D_* contribute to *P* in a similar fashion for both *Homo sapiens* and *Macaca mulatta*. For both species one quart of the phenotype is given by the static component and three quart by the dynamic component: 
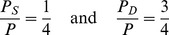



The multilevel model attempts to describe the difference among *P*_S_ and *P*_D_ of different individuals by modelling their average metabolic profiles. The separation observed among individuals arises by differences in the mean concentration levels (across the 30–40 days span of the urine collection) of the urinary metabolites of each different individual. From Figure 2 it is clear that the differences in *P_S_* between subjects of the same gender are smaller than the differences among individuals of different genders. This indicates that *P_S_* is mostly related to gender (biologically an intrinsic characteristic) as previously observed in [1] and [11].

The models for both species contain very similar panels of metabolites whose average concentrations are responsible for differences in *P_S_* (see Figure 2). The multilevel model is dominated, among others, by creatinine, whose levels are known to be different in males and females. The levels of creatinine relate to the lean body mass (muscle mass), which is in general larger in men [23]. The creatine/creatinine biosynthesis is conserved in all vertebrates [24] and is linked to the arginine biosynthesis pathway which is universally present in all three domains of life [25].

For *Homo sapiens* one cannot exclude *a priori* that different dietary habits could modulate these patterns of variations, but as monkeys were kept generally on a standardized diet (*i.e*. they received the same food day by day) and as the biological machinery underlying these metabolites is conserved (the spectrum mainly includes central metabolism) the outcome may be extrapolated to humans. These findings lead us to speculate that for *P_S_* extrinsic factors contribute little compared to intrinsic factors.

On the basis of this, Equation (3) can be re-written as
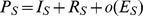
where with the notation *o(E_S_)* we indicate that the contribution of *E_S_* (and its interactions) is much smaller than the other terms. By comparing results obtained for *Homo sapiens* and *Macaca mulatta*, this relationship holds true for both species.

The NMR-based urinary metabolic phenotype is high-dimensional in nature, arising from hundreds to thousands of molecules, but multilevel analysis showed to be a convenient tool to reduce it to two-three dimensions. The low-dimensional representation can be easily used to detect aberrant static phenotypes. With reference to Figure 2 we can observe two cases of such deviations. For *Homo sapiens* one of the male individuals deviates from the region (in this case bi-dimensional) of the static phenotype occupied by male subjects. Interestingly, this male subject is not an outlier anymore if TMAO is not considered in the analysis (not shown). This means that for this subject the TMAO concentration markedly differ, at the mean level, from that of other (male) subjects. In principle the mean level should not be influenced by sporadic consumption of fish but it could be affected, for instance, by a fish-rich diet. As both situations were excluded, we speculate that this subject may suffer (or have suffered) from some sort of alteration of the TMAO metabolism/microbiome composition.

For *Macaca mulatta* animals, one of the female individual falls in a remote empty region of the phenotype landscape, well away from other subjects. This indicates that both the humans and monkey subjects are, in terms of mean metabolite concentration levels, different from the others.

### Multilevel analysis highlights similarities in the dynamic urinary phenotype of *Homo sapiens* and *Macaca mulatta*

For *Homo sapiens* the individual dynamic variation explained per individual is quite variable, ranging from 31% to 91%. This measure provides information about how well the dynamic phenotype of a subject conforms to the multilevel model; this means that subjects with similarly high values of dynamic variation explained have qualitatively similar dynamic phenotypes. Subjects with lower values conform less well to the model, indicating that their *P_D_* is qualitatively different. The large range of values observed for the *P_D_* variability explained by the multilevel model indicates that *P_D_* is qualitatively different among different subjects. Notably, the same high degree of variability is also observed in *Macaca mulatta*, showing that the individual metabolic profile is robust and can be used for personalized treatments. As can be seen in Figure 5 (Panel A), the contribution from TMAO, creatine/creatinine, phenylactylglicine, mHPPA, and 1-methylhistidine appears also in the human dynamic model; therefore different dynamics of these metabolites are responsible for the different *P_D_* in individuals. The dynamic model for the *Macaca mulatta* is also dominated by TMAO, acetate and creatine thus partially replicating the same pattern of variation observed for humans. Most of these metabolites have a role in central metabolism, which is presumably tightly regulated. However, their dynamic nature cannot be attributed to extrinsic variation: the multilevel models are indeed similar for both species but influence of extrinsic factors on the dynamic the metabolites responsible for *P_D_* can be excluded. Following a line of reasoning similar to the case of the static phenotype we can speculate that also for *P_D_* extrinsic factors are small with respect to intrinsic factor and Equation (3) can be re-written as 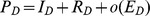
and also this relationship holds true for both species.

The variation explained by the dynamic model (see Materials and Methods) is a measure of the diversity of *P_D_* of different subjects. For one of the monkeys the explained dynamic is 49%, considerably lower than that explained for other monkeys and, incidentally, this animal is the same who is an outlier with respect to *P_S_*. *A posteriori* we attributed this to either an underlying diseased status which went unrecognized or undetected at the time of urine collection or to a different social status of the individual that could have resulted in a condition of stress.

The human male outlier in the static model previously discussed has a low *P_D_* diversity explained by the model (48%), but not as low as the female subject (barely 30%), that had normal *P_D_*.

### Evidence of individual metabolic phenotype in *Macaca mulatta*

**Figure 7:**
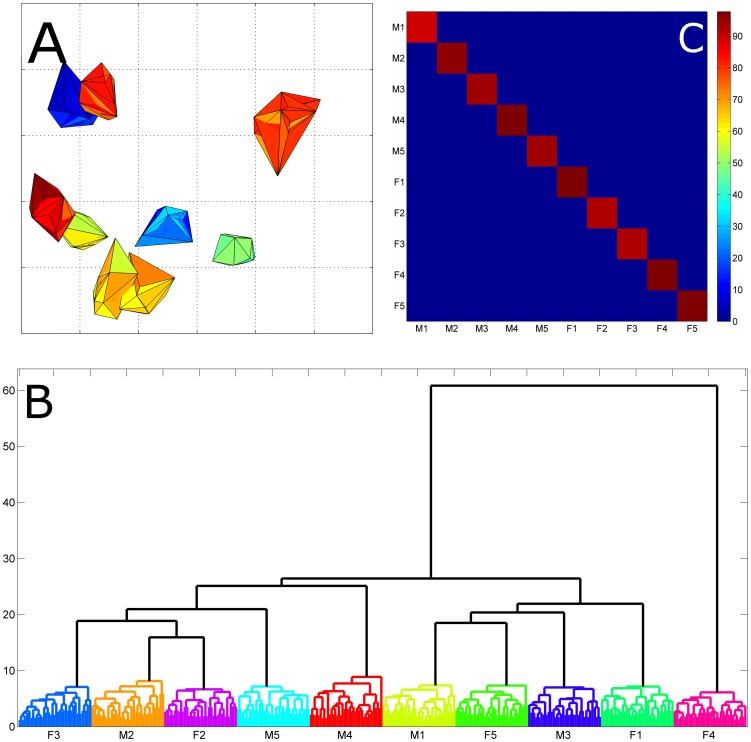
Exploratory and predictive analysis of the *Macaca mulatta* data. Panel A) Two-dimensional projection of the ^1^H NMR spectral buckets into the PCA/CA subspace in the three most significant dimensions. Each convex hull represents an animal-specific cluster of points (*i.e*. 30 NMR spectra). This figure parallels, for the monkey case the Figure 1 in [1]. To enhance clarity only 9 monkeys are shown, removing the outlier monkey. Panel B) Dendrogram plot relative to hierarchical cluster analysis (HCA). The dendrogram represents the inter-sampling distances in the 8-dimensional discriminant space of the PCA-CA components. (*M*: male monkey, *F*: female monkey). Female monkey F4 is clearly an outlier. This figure parallels, for the monkey case the Figure 2 in [1]. Panel C) Classification accuracy for each monkey using the PCA-CA-KNN method. The P-value (calculated by means of a permutation test [18] was <0.01 for every monkey.

To our knowledge the existence of individual metabolic phenotypes for species other than *Homo sapiens* was, in contrast to genotypes, hitherto never investigated. The question was whether also *Macaca mulatta* possesses an individual phenotype as found for humans in [1], [9]. To ascertain this and for sake of comparability with the previous studies we re-analyzed the *Macaca mulatta* data set with the same statistical approach used in [1], [9] where the existence of an individual metabolic phenotype was shown for humans. We were able to reproduce the findings in [1], [9] showing that, as for humans, each urine spectrum carries highly donor-specific traits able to provide a fingerprint characteristic for each animal; this fingerprint allows correct identifications of a donor animal from unknown urine samples not previously included in the statistical model (PCA-CA-KNN) used for the discriminatory/predictive analysis. Results of the analysis are shown in Figure 7 (Panel A to C) in analogy with Figures 1, 2 and 3 in [1]. Classification results for each monkey are given in Table S2 in File S1.

We comment here that genetic likeliness could not be taken into account in this analysis: it may well be that the genetic similarity of the monkeys in the controlled environment is more similar than the human subjects. This makes the observation that individuals can be recognized based on their urine spectrum even more remarkable together with the fact that, in contrast to humans, the monkeys were all fed the same diet. These findings provide evidence that individual metabolic phenotypes exist and its subject-specificity is not measurably influenced by external factors.

### The nature of the individual metabolic phenotype

The findings discussed above show that a great degree of diversity observed in *P* of both *Homo sapiens* and *Macaca mulatta* can be attributed to differences in both the static and dynamic part. For both species we have shown that these two components sum to the overall metabolic phenotype with different relative contributions of 1/4 and 3/4 respectively.

In this framework it is clear that similarities/dissimilarities among individual metabolic phenotypes observed in previous studies (such as [1], [9]) reflect similarities/dissimilarities due to static differences between individuals which are constant in time (like gender and genotype) and from dynamic differences which are subject specific (like biorhythms and microbiome).

In a recent paper Nicholson *et al.* [26] attempt to investigate the contributing factors to the variability observed in urine and blood between subjects (thus differences in *P_S_* although this was defined with one or at maximum two samples per subjects rather than with 30 like in our study) and found that ∼50% of the variation is accountable to stable variation, comprising familial and environmental variation.

We observed substantial similarity in *P_S_* and *P_D_* for the two species and provided evidence that for both the phenotypic inequality *P_S_<P_D_* holds. Moreover we have shown that individuals of both species possess an individual discriminant urinary phenotype, as indicated by the fact that discrimination among different animals is possible with accuracies ranging between 85% and 100%. This very high recognition accuracy, although slightly less than that found in the human dataset, which ranged between 96% and 100% [1], occurs despite the standardized environment (including diet) where the monkeys are kept.

In particular the setting of this study allowed a more thorough discussion of the contribution of the diet in the makeup of the urinary metabolic profile as far as it concerns its component captured by NMR profiling.

It has been recently postulated that dietary shift may have contributed to phenotypic changes seen in modern humans as compared with non-human primates [27] and there is evidence of selection for certain genomic signatures by dietary shift in modern humans [28]–[31] compared to non-human primates. While [6] and [7] report strong association between diet and phenotypes, Winnike and co-workers [8] reported that diet may play only a minor role in the individual phenotype, a result substantiate by [32], that showed that also under restricted environmental conditions, the largest source of variability in urine metabolome(s) was attributable to technical variation, rather than to biological variables, meals, or time of day [32]. Bernini and co-workers [9] report anecdotic evidence that metabolic phenotype was unaltered upon major changes in diet and lifestyle. The group of healthy volunteers in the *Homo sapiens* project [1], [9] resulted to be a rather uniform cohort of individuals (see Table S1 in File S1); the different dietary habits could be probably regarded as the major contributing environmental factor. In the case of the *Macaca mulatta* study, in contrast, differences in dietary habits and food intake were minimized by keeping the animals on a standardized dietary regime and environment. For the monkeys, the dietary regime was standardized across the animals such that for each day the diet was the same and this enables eliminating possibly diet-induced variations. How this is possible is clear from Equation 4 (and associated references). The static part of the model describes the deviation of each individual from the overall average level: the diet being the same for all subjects, its effect cancels out because the overall mean is subtracted before considering the mean of each subject. Analogously, the dynamic part of the model describes the individual responses across time, in deviation from the individual-specific mean across time. In contrast, for the humans, the diet differed between days and subjects. This implies that diet effects do not cancel out across human subjects, and thus it is impossible to distinguish different dietary habitudes from intrinsic factors as sources of the observed differences.

On the basis of this we can infer that dietary habits play a minor role to the shaping of the urinary NMR metabolic phenotype, thus substantiating the results in [8]. Moreover this observation corroborates the findings of [1], [9] excluding that the discriminative power carried by the individual NMR urinary phenotype could be just a result of different dietary habits of subjects.

Our results were derived on a cohort of 31 individuals of both genders, all of them healthy subjects, which we may consider a representative sample of a western European/Caucasian population. Due to the limited size and to the lack of stratification this cohort may not be fully representative of the diversity observed in the overall human population (both at the genomic level and at the level of dietary habits imposed by geographical segregation); this caveat being also true in the case of the *Macaca mulatta* population sample. Nonetheless, our findings point mostly to fundamental metabolic and biochemical processes as the key drivers of the shaping of the urinary metabolic phenotype and suggest the validity of the results here shown to hold also for a larger population, pending experimental confirmation.

Some of the metabolites highlighted by the multilevel analysis could be associated to some extent to gut microflora (mHPPA, PAG). Evidence has been brought about the possible role of gut microflora in shaping the urinary metabolic phenotype [33]-[35] but there is also evidence that genetically related subjects tend to share more similar gut microflora that unrelated subjects [36]. In this respect it is difficult to decide whether gut microflora should be considered an extrinsic or rather an intrinsic factor in the phenotype equation. Nonetheless, the observation that the NMR-based urinary metabolic phenotype arises mostly from intrinsic factors strengthens the idea that the metabolic phenotype can be advantageous for improving personalized therapy and nutrition, enhancing pharmacometabonomics approaches to better predicting and assessing both drug efficacy and toxicity and understanding disease aetiology.

## Supporting Information

Checklist S1 **ARRIVE Checklist.**
(DOC)

File S1 Table S1, S2 and S3 and a supporting note N1 about NMR sample preparation.
(DOCX)

## References

[1] Assfalg M, Bertini I, Colangiuli D, Luchinat C, Schafer H, et al (2008) Evidence of different metabolic phenotypes in humans. Proc Natl Acad Sci U S A 105: 1420–1424. 18230739 10.1073/pnas.0705685105 PMC2234159

[2] Suhre K, Gieger C (2012) Genetic variation in metabolic phenotypes: study designs and applications. Nature reviews genetics 13: 759–769. 10.1038/nrg3314 23032255

[3] Duarte NC, Becker SA, Jamshidi N, Thiele I, Mo ML, et al (2007) Global reconstruction of the human metabolic network based on genomic and bibliomic data. Proceedings of the National Academy of Sciences 104: 1777–1782. 10.1073/pnas.0610772104 PMC1794290 17267599

[4] Duarte NC, Palsson BØ, Fu P (2004) Integrated analysis of metabolic phenotypes in Saccharomyces cerevisiae. BMC genomics 5: 63. 15355549 10.1186/1471-2164-5-63 PMC520746

[5] Feuerbach L (1862) "Das Geheimnis des Opfers, oder, Der Mensch ist, was er isst”. Gesammelte Werke. 22: 26–52.

[6] Stella C, Beckwith-Hall B, Cloarec O, Holmes E, Lindon JC, et al (2006) Susceptibility of human metabolic phenotypes to dietary modulation. J Proteome Res 5: 2780–2788. 17022649 10.1021/pr060265y

[7] Holmes E, Loo RL, Stamler J, Bictash M, Yap IKS, et al (2008) Human metabolic phenotype diversity and its association with diet and blood pressure. Nature 453: 396–400. 18425110 10.1038/nature06882 PMC6556779

[8] Winnike JH, Busby MG, Watkins PB, O'Connell TM (2009) Effects of a prolonged standardized diet on normalizing the human metabolome. American Journal of Clinical Nutrition 90: 1496. 19864408 10.3945/ajcn.2009.28234 PMC2777465

[9] Bernini P, Bertini I, Luchinat C, Nepi S, Saccenti E, et al (2009) Individual human phenotypes in metabolic space and time. J Proteome Res 8: 4264–4271. 19527021 10.1021/pr900344m

[10] Yousri NA, Kastenmüller G, Gieger C, Shin S-Y, Erte I, et al.. (2014) Long term conservation of human metabolic phenotypes and link to heritability. Metabolomics: 1–13. 10.1007/s11306-014-0629-y PMC4145193 25177233

[11] Jansen JJ, Hoefsloot HCJ, van der Greef J, Timmerman ME, Smilde AK (2005) Multilevel component analysis of time-resolved metabolic fingerprinting data. Analytica chimica acta 530: 173–183.

[12] Reinberg A (1974) Des rythmes biologiques à la chronobiologie: Gauthier-Villars.

[13] Lestienne R (1988) From physical to biological time. Mechanisms of ageing and development 43: 189–228. 3205059 10.1016/0047-6374(88)90032-2

[14] Angeli A (1974) Circadian ACTH-adrenal rhythm in man. Chronobiologia 1: 253. 4377960

[15] Moore RY, Klein DC (1974) Visual pathways and the central neural control of a circadian rhythm in pineal serotonin N-acetyltransferase activity. Brain Research 71: 17–33. 4595289 10.1016/0006-8993(74)90188-7

[16] Timmerman ME (2006) Multilevel component analysis. British Journal of Mathematical and Statistical Psychology 59: 301–320. 17067414 10.1348/000711005X67599

[17] Brok H, Tekoppele J, Hakimi J, Kerwin J, Nijenhuis E, et al (2001) Prophylactic and therapeutic effects of a humanized monoclonal antibody against the IL-2 receptor (DACLIZUMAB) on collagen-induced arthritis (CIA) in rhesus monkeys. Clinical & Experimental Immunology 124: 134–141. 11359452 10.1046/j.1365-2249.2001.01487.x PMC1906026

[18] Szymańska E, Saccenti E, Smilde AK, Westerhuis JA (2012) Double-check: validation of diagnostic statistics for PLS-DA models in metabolomics studies. Metabolomics 8: 3–16. 22593721 10.1007/s11306-011-0330-3 PMC3337399

[19] Searle SR, Casella G, McCulloch CE (2009) Variance components: John Wiley & Sons.

[20] Jolliffe IT (2002) Principal component analysis: Wiley Online Library.

[21] Van Den Berg RA, Hoefsloot HCJ, Westerhuis JA, Smilde AK, Van Der Werf MJ (2006) Centering, scaling, and transformations: improving the biological information content of metabolomics data. BMC genomics 7: 142. 16762068 10.1186/1471-2164-7-142 PMC1534033

[22] Timmerman ME, Kiers HAL, Smilde AK, Ceulemans E, Stouten J (2009) Bootstrap confidence intervals in multi-level simultaneous component analysis. British Journal of Mathematical and Statistical Psychology 62: 299–318. 18086338 10.1348/000711007X265894

[23] Baxmann AC, Ahmed MS, Marques NC, Menon VB, Pereira AB, et al (2008) Influence of muscle mass and physical activity on serum and urinary creatinine and serum cystatin C. Clinical Journal of the American Society of Nephrology. 3: 348–354. 10.2215/CJN.02870707 PMC2390952 18235143

[24] Wyss M, Kaddurah-Daouk R (2000) Creatine and creatinine metabolism. Physiological Reviews 80: 1107. 10893433 10.1152/physrev.2000.80.3.1107

[25] Peregrin-Alvarez JM, Tsoka S, Ouzounis CA (2003) The phylogenetic extent of metabolic enzymes and pathways. Genome research 13: 422. 12618373 10.1101/gr.246903 PMC430287

[26] Nicholson G, Rantalainen M, Maher AD, Li JV, Malmodin D, et al.. (2011) Human metabolic profiles are stably controlled by genetic and environmental variation. 10.1038/msb.2011.57 PMC3202796 21878913

[27] Babbitt CC, Warner LR, Fedrigo O, Wall CE, Wray GA (2010) Genomic signatures of diet-related shifts during human origins. Proceedings of the Royal Society B: Biological Sciences. 10.1098/rspb.2010.2433 PMC3049039 21177690

[28] Perry GH, Dominy NJ, Claw KG, Lee AS, Fiegler H, et al (2007) Diet and the evolution of human amylase gene copy number variation. Nature genetics 39: 1256–1260. 17828263 10.1038/ng2123 PMC2377015

[29] Enattah NS, Sahi T, Savilahti E, Terwilliger JD, Peltonen L, et al (2002) Identification of a variant associated with adult-type hypolactasia. Nature genetics 30: 233–237. 11788828 10.1038/ng826

[30] Tishkoff SA, Reed FA, Ranciaro A, Voight BF, Babbitt CC, et al (2006) Convergent adaptation of human lactase persistence in Africa and Europe. Nature genetics 39: 31–40. 17159977 10.1038/ng1946 PMC2672153

[31] Kelley JL, Swanson WJ (2008) Dietary change and adaptive evolution of enamelin in humans and among primates. Genetics 178: 1595. 18245370 10.1534/genetics.107.077123 PMC2278081

[32] Kim K, Mall C, Taylor SL, Hitchcock S, Zhang C, et al (2014) Mealtime, Temporal, and Daily Variability of the Human Urinary and Plasma Metabolomes in a Tightly Controlled Environment. PloS one 9: e86223. 24475090 10.1371/journal.pone.0086223 PMC3901684

[33] Calvani R, Miccheli A, Capuani G, Miccheli AT, Puccetti C, et al (2010) Gut microbiome-derived metabolites characterize a peculiar obese urinary metabotype. International Journal of Obesity 34: 1095–1098. 20212498 10.1038/ijo.2010.44

[34] Li M, Wang B, Zhang M, Rantalainen M, Wang S, et al (2008) Symbiotic gut microbes modulate human metabolic phenotypes. Proceedings of the National Academy of Sciences 105: 2117–2122. 10.1073/pnas.0712038105 PMC2538887 18252821

[35] Nicholson JK, Holmes E, Wilson ID (2005) Gut microorganisms, mammalian metabolism and personalized health care. Nature Reviews Microbiology 3: 431–438. 15821725 10.1038/nrmicro1152

[36] Zoetendal EG, Akkermans AD, Akkermans-van Vliet WM, de Visser JAG, de Vos WM (2001) The host genotype affects the bacterial community in the human gastronintestinal tract. Microbial Ecology in Health and Disease 13: 129–134.

