# Supplementary material for: Of Monkeys and Men: A Metabolomic Analysis of Static and Dynamic Urinary Metabolic Phenotypes in Two Species
Source: PLoS One. 2014 Sep 15;9(9):e106077. doi: 10.1371/journal.pone.0106077 (PMC4164446; doi:10.1371/journal.pone.0106077)
Supplement: File S1 — Table S1, S2 and S3 and a supporting note N1 about NMR sample preparation. (DOCX) [file pone.0106077.s002.docx]

**Supporting Information FILE S1**

# Of men and monkeys: a metabolomic analysis of the static and dynamic urinary metabolic phenotypes in two species

### Edoardo Saccenti^1,2,§^, Leonardo Tenori ^3,8^, Paul Verbruggen^4^, Marieke E. Timmerman^5^, Jildau Bouwman^6^, Jan van der Greef^6,7^, Claudio Luchinat^3,9^, Age K. Smilde^2^

^1^Laboratory of Systems and Syntetic Biology, Wageningen University and Research Center, Dreijenplein 10, 6703 HB, Wageningen The Netherlands

^2^Biosystems Data Analysis Group, University of Amsterdam^,^ Science Park 904, 1098 XH Amsterdam, The Netherlands

^2^Laboratory of Systems and Syntetic Biology, Wageningen University and Research Center, Dreijenplein 10, 6703 HB, Wageningen The Netherlands

^3^Center for Magnetic Resonance, University of Florence, via Luigi Sacconi 6, Sesto Fiorentino, Italy

^4^Nuclear Organization Group, University of Amsterdam^,^ Science Park 904, 1098 XH Amsterdam, The Netherlands

^5^Heymans Institute, University of Groningen, Grote Kruisstraat 2/1, 9712TS Groningen, The Netherlands

^6^TNO, Utrechtseweg 48, 3704HE, Utrecht, The Netherlands

^7^Sino-Dutch Centre for preventive and personalized medicine, Utrechtseweg 48, 3704HE, Utrecht, The Netherlands

^8^FiorGen Foundation, via Luigi Sacconi 6, Sesto Fiorentino, Italy

^9^Department of Chemistry, University of Florence, via della Lastruccia 3, Sesto Fiorentino, Italy

^§^Corresponding author: Edoardo Saccenti edoardo.saccenti@wur.nl

**Supporting Note S1**

**On the NMR sample processing**

We want to stress again that the two data sets was analyzed independently from each other. We understand that this was not clear in the first version of the paper, and we have now spent more effort in clarifying this point all-along the text. In practice, no comparison was made between the experimental data but only the results of the same statistical analysis were compared and discusses. As such, the observed differences are not attributable to different experimental settings and procedures. This high level data fusion approach is the only possible to overcome the problem that being this study a re-analysis of existing and published data, no new experiments were made possible.

We appreciate that the lyophilization process can in principle lead to a loss of some volatile compounds present in urine. Nevertheless this is not a matter of concern because both studies (and of consequence the present study) focused on soluble urine metabolites. Whether the focus had been on the volatiles, NMR would have not been a suitable analytical platform to measure such compounds: for this case the method of election is mass spectrometry, namely GC-MS ([Carrola, Rocha et al. 2010](#_ENREF_1); [Rocha, Caldeira et al. 2012](#_ENREF_4); [Loureiro, Duarte et al. 2014](#_ENREF_3)).

Moreover, lyophilization is used to concentrate sample and to possibly recover signal from metabolites in low abundance due to limited quantity of sample obtainable from the animals.

Anyway, even if slightly alteration of the metabolic profile of a urine sample would be induced by the different sample preparation, the magnitude of these changes is not larger enough to hidden or confuse the individual fingerprints. This has been shown in the literature ([Lauridsen, Hansen et al. 2007](#_ENREF_2)): in the comparison of several different protocols for urine samples storage and preparation, including lyophilization, it was found that the major source of variation among samples remain that due to the urine sample origin, that is the *inter-individual variation,* rather than to different sample handling and/or preparation.

Finally, an inter-laboratory comparison ([Ward, Baker et al. 2010](#_ENREF_5)) has shown that, in contrast with GC-MS, ^1^H-NMR metabolite fingerprinting is a robust technique that allow unbiased comparison of data collected in different laboratories with instruments operating at different field strength (400, 500, and 600 MHz).

**REFERENCES**

Carrola, J., C. M. Rocha, et al. (2010). "Metabolic signatures of lung cancer in biofluids: NMR-based metabonomics of urine." Journal of Proteome Research **10**(1): 221-230.

Lauridsen, M., S. H. Hansen, et al. (2007). "Human Urine as Test Material in 1H NMR-Based Metabonomics:  Recommendations for Sample Preparation and Storage." Analytical chemistry **79**(3): 1181-1186.

Loureiro, C. C., I. F. Duarte, et al. (2014). "Urinary metabolomic changes as a predictive biomarker of asthma exacerbation." The Journal of allergy and clinical immunology **133**(1): 261-263.e265.

Rocha, S. M., M. Caldeira, et al. (2012). "Exploring the human urine metabolomic potentialities by comprehensive two-dimensional gas chromatography coupled to time of flight mass spectrometry." Journal of Chromatography A **1252**: 155-163.

Ward, J. L., J. M. Baker, et al. (2010). "An inter-laboratory comparison demonstrates that [1 H]-NMR metabolite fingerprinting is a robust technique for collaborative plant metabolomic data collection." Metabolomics **6**(2): 263-273.

**Supporting Table S1**

**Anthropomorphic characteristics of the human participants**

|  |  |  |  |  |  |  |  |
| --- | --- | --- | --- | --- | --- | --- | --- |
| \| **Code** \| **Weigth (Kg)** \| **Heigth (m)** \| **Gender** \| **Age (at analysis)** \| **Nationality** \| **Smoking Status** \| **Diet** \| \| --- \| --- \| --- \| --- \| --- \| --- \| --- \| --- \| \|  \|  \|  \|  \|  \|  \|  \|  \| \| **AI** \| 55 \| 1.63 \| F \| 31 \| Italian \| not smoker \| Mediterranean \| \| **AO** \| 75 \| 1.54 \| M \| 33 \| Italian \| not smoker \| Mediterranean \| \| **AR** \| 80 \| 1.78 \| M \| 34 \| Italian \| ex smoker \| Mediterranean \| \| **AS** \| 80 \| 1.77 \| M \| 30 \| Italian \| not smoker \| Mediterranean \| \| **AU** \| 95 \| 1.8 \| M \| 36 \| Italian \| not smoker \| Mediterranean \| \| **AW** \| 65 \| 1.82 \| M \| 30 \| Italian \| not smoker \| Mediterranean \| \| **BC** \| 55 \| 1.66 \| F \| 28 \| Italian \| not smoker \| Mediterranean \| \| **BF** \| 87 \| 1.85 \| M \| 36 \| Italian \| not smoker \| Mediterranean \| \| **BG** \| 65 \| 1.61 \| F \| 30 \| Italian \| not smoker \| Mediterranean \| \| **BH** \| 75 \| 1.67 \| F \| 37 \| Italian \| not smoker \| Mediterranean \| \| **BI** \| 63 \| 1.78 \| F \| 37 \| Italian \| not smoker \| Mediterranean \| \| **BQ** \| 70 \| 1.75 \| M \| 26 \| Italian \| not smoker \| Mediterranean \| \| **BS** \| 40 \| 1.6 \| F \| 26 \| Italian \| not smoker \| Mediterranean \| \| **BT** \| 60 \| 1.59 \| F \| 27 \| Italian \| not smoker \| Mediterranean \| \| **BU** \| 62 \| 1.72 \| M \| 55 \| Italian \| not smoker \| Mediterranean \| \| **BV** \| 62 \| 1.79 \| M \| 21 \| Italian \| not smoker \| Mediterranean \| \| **BX** \| 53 \| 1.6 \| F \| 24 \| Italian \| not smoker \| Mediterranean \| \| **BZ** \| 45 \| 1.53 \| F \| 26 \| Italian \| not smoker \| Mediterranean \| \| **TA** \| 64 \| 1.77 \| F \| 35 \| Italian \| not smoker \| Mediterranean \| \| **TB** \| 65 \| 1.77 \| F \| 35 \| Italian \| not smoker \| Mediterranean \| \| AD \| 55 \| 1.68 \| F \| 33 \| Italian \| smoker \| Mediterranean \| \| AF \| 55 \| 1.75 \| F \| 50 \| Italian \| not smoker \| Mediterranean \| \| AG \| 52 \| 1.6 \| F \| 28 \| Italian \| not smoker \| Mediterranean \| \| AH \| 52 \| 1.58 \| F \| 34 \| Italian \| not smoker \| Mediterranean \| \| AP \| 80 \| 1.76 \| M \| 32 \| Italian \| not smoker \| Mediterranean \| \| AT \| 70 \| 1.8 \| M \| 33 \| Italian \| not smoker \| Mediterranean \| \| AX \| 93 \| 1.81 \| M \| 29 \| Italian \| not smoker \| Mediterranean \| \| AZ \| 63 \| 1.7 \| F \| 30 \| Italian \| not smoker \| Mediterranean \| \| BD \| 53 \| 1.7 \| F \| 29 \| Italian \| not smoker \| Mediterranean \| \| BE \| 70 \| 1.8 \| M \| 33 \| Italian \| not smoker \| Mediterranean \| \| BK \| 71 \| 1.72 \| M \| 47 \| Italian \| not smoker \| Mediterranean \| |  |  |  |  |  |  |  |

**Supporting Table S2**

**The Individual Single Vote Scores the monkey data set.**

| **Monkey ID** | **R %** | ***P-val*** |
| --- | --- | --- |
| **MM01** | 85.6 | <0.001 |
| **MM02** | 95.0 | <0.001 |
| **MM03** | 92.8 | <0.001 |
| **MM04** | 97.0 | <0.001 |
| **MM05** | 96.4 | <0.001 |
| **MF01** | 100.0 | <0.001 |
| **MF02** | 93.0 | <0.001 |
| **MF03** | 99.3 | <0.001 |
| **MF04** | 96.5 | <0.001 |

**Supporting Table S3**

| ANOVA | Analysis of variance |
| --- | --- |
| BMI | Body mass index |
| BPRC | Biomedical Primate Research Centre |
| CA | Canonical analysis |
| CI(S) | Confidence interval(s) |
| *E* | Extrinsic contributing factor(s) to the phenotype |
| KNN | *k*-the nearest neighbour |
| *I* | Intrinsic contributing factor(s) to the phenotype |
| MANOVA | Multivariate Analysis of Variance |
| mHPPA | meta-hydroxyphenyl-propionic acid |
| MSCA | Multilevel simultaneous component analysis |
| NMR | Nuclear magnetic resonance (spectroscopy) |
| *P* | (urinary) metabolic phenotype |
| *P_D_* | Dynamic part of the phenotype |
| *P_S_* | Static part of the phenotype |
| PCA | Principal component analysis |
| PAG | Phenyl-acetyl-glycine |
| SCA | Simultaneous component analysis |
| TMAO | Trimethylamine N-oxide |
| TSP | sodium trimethylsilyl-[2,2,3,3,-2H4]-1-propionate |
